# Analysis of news media reports of suicides and attempted suicides during the COVID-19 lockdown in India

**DOI:** 10.1186/s13033-020-00422-2

**Published:** 2020-12-03

**Authors:** Soumitra Pathare, Lakshmi Vijayakumar, Tanya Nicole Fernandes, Manisha Shastri, Arjun Kapoor, Deepa Pandit, Isha Lohumi, Somidha Ray, Arti Kulkarni, Palak Korde

**Affiliations:** 1 Centre for Mental Health Law and Policy, Indian Law Society, Pune, India; 2 Sneha - Suicide Prevention Centre, Voluntary Health Services, Chennai, Tamil Nadu India

**Keywords:** Suicide, Attempted suicides, COVID-19, Lockdown, Reports, India

## Abstract

**Background:**

Based on previous experience there is justifiable concern about suicidal behaviour and news media reporting of it during COVID-19 pandemic.

**Methods:**

This study used a systematic search of online news media reports (versions of newspapers, magazine and other digital publications) of suicidal behaviour during India’s COVID-19 lockdown and compared it to corresponding dates in 2019. Data was gathered using a uniform search strategy from 56 online news media publications 24 March to 3 May for the years 2019 and 2020 using keywords, suicide, attempted suicide, hangs self and kills self. Demographic variables and methods used for suicide were compared for suicide and attempts between the 2 years using chi-squared tests (χ^2^).

**Results:**

There were online news media reports of 369 cases of suicides and attempted suicides during COVID lockdown vs 220 reports in 2019, a 67.7% increase in online news media reports of suicidal behaviour. Compared to 2019, suicides reported during lockdown were significantly older (30 vs 50 years, p < 0.05), men (71.2% vs 58.7%; p < 0.01), married (77.7% vs 49%; p < 0.01) and employed (82.9% vs 59.5%; p < 0.01). During the lockdown, significantly more suicides were by hanging (64.4% vs 42%), while poisoning (8.5% vs 21.5%) and jumping in front of a train (2% vs 9.4%) (p < 0.05) were significantly reduced. Comparison of COVID and non-COVID groups showed that online news media reports of COVID cases of suicide and attempted suicide were significantly more likely to be men (84.7% vs 60.4%; p < 0.01), older (31–50 years 52.9% vs 25.8%; p < 0.01) employed (91.5% vs 64.3%; p < 0.01), had poor mental (40.1% vs 20.8%; p < 0.01) and poor physical health (24.8% vs 7.9%;11.8, p < 0.01).

**Conclusion:**

Increase in online news media reports of suicides and attempts during COVID-19 lockdown may indicate an increase in journalists’ awareness about suicide or more sensational media reporting or may be a proxy indicator of a real community increase in suicidal behaviour. It is difficult to attribute changes in demographic profile and methods used only to changes in journalists’ reporting behaviour and should be further explored. We therefore call upon the Government of India to urgently release national suicide data to help devise a comprehensive suicide prevention strategy to address COVID-19 suicidal behaviour.

## Background

Suicides and attempted suicides are an important public health concern in the current COVID-19 pandemic. There is justifiable concern that suicide and attempted suicides may increase during the COVID pandemic [1, 2]. This is based on studies showing increased suicides in USA during the 1918 influenza epidemic [3] and amongst elderly after SARS epidemic attributed to the breakdown of social networks, limited access to healthcare, fear of contracting the virus, social disengagement, mental stress and anxiety and fear of being a burden on their families [4, 5]. Single case studies on suicides and attempted suicides during the current COVID pandemic have been reported [6] but there is no systematic data as yet on suicide and attempted suicide.

Before the pandemic, in 2016, it is estimated that India with only 17.8% of global population accounted for 36·6% and 24.3% of the global suicide deaths in women and men, respectively [7]. Official data on suicides in India is recorded by the National Crime Record Bureau (NCRB) by collating information from police records. However, NCRB data has been shown to significantly underestimate suicide rates due to under-reporting of cases and this data is usually made available after a significant delay of between 12 and 24 months. Furthermore, NCRB releases summary annual data rather than weekly or monthly data to analyse trends and NCRB does not keep any record of attempted suicides.

India recorded its first COVID case on 30th January 2020 [8] and as the number of cases increased, Government of India progressively instituted a range of measures starting with suspending travel from certain countries, instituting medical screening for international travellers and finally a complete nation-wide lock-down from 24 March 2020 to 14 April 2020 which was subsequently extended to 3 May 2020. This is possibly one of the largest lockdowns ever recorded in history of the country affecting 1.3 billion citizens and also one of the severest in the world with thousands of workers walking back to their villages in the absence of public transport and loss of livelihoods, jobs and accommodation in India’s large cities [9].

As the lockdown started, we noticed newspapers reporting suicides, linking them to the COVID pandemic and the lockdown. We were interested in understanding whether media reporting of suicides and attempted suicides in India changed during these 40 days of lockdown from 24 March 2020 to 3 May 2020. We therefore, planned a systematic search of English language online news media (referred to as news reports subsequently in the paper) for reports of suicide and attempted suicide during the lockdown and compared them to news reports of suicides and attempted suicides for the same dates in 2019 in an attempt to understand how the COVID related lockdown may have impacted suicides and attempted suicides reporting in Indian news publications.

## Methods

To estimate the number of news reports of cases of suicide and attempted suicide during the COVID-19 lockdown in India from 24 March to 3 May 2020, we identified, sourced and gathered data as described below.

From the date of start of lockdown on 24 March 2020, a daily Google News search was carried out to identify relevant articles reporting deaths by suicide or attempted suicide. From this search, 209 relevant articles from 65 online English language publications were identified. The publications included some of the online versions of the highest read daily newspapers across the country such as Times of India, Hindustan Times, The Hindu, The Indian Express, The New Indian Express, The Tribune and The Telegraph, each with minimum monthly average readership of 1.5 million according to the Indian Readership Survey 2019 [10]. From the Google News search, we also found that magazines such as Outlook India and the India Today frequently reported cases of suicide or attempted suicide. The 65 publications also had articles from online news websites (digital only publications) such as The Wire, The Quint, The Caravan and The Scroll that have a wide online reach. In addition, there were regional language news media with English editions among the publications that were retrieved from the Google News search for example Arunachal24, Mathrubhumi, The Orissa Post and Telangana Today. With this initial Google News search, we were able to identify a list of 65 publications, each reporting at least one report of suicide or attempted suicide during the period of 24 March to 3 May 2020.

We then conducted a comprehensive search across these pre-identified online publications. From the 65 publications, nine were excluded as they were not India-based, not in English or were not considered legitimate sources of news by Google, leaving 56 English online publications with pan-India news coverage. The 56 publications comprised of online websites of national-level newspapers (n = 16) TV news channels (n = 5), magazines (n = 4) and digital publications (n = 22). There were also online English language versions of regional language newspapers (n = 7) and regional digital only publications (n = 2). A majority of the articles were sourced from online websites of newspapers with the highest average readership in the country. A uniform search strategy was applied using the following keywords: suicide, attempted suicide, hangs self and kills self for the lockdown dates of 24 March 2020 to 3 May 2020 and using the name of the publication in the search terms sequentially. All Google hits with this search strategy were manually scrutinized by a researcher to find relevant suicide articles. The criteria for including an article were: (i) the article provided enough unique identifiers of the individual dying of suicide/attempting suicide to ensure we did not count any individual multiple times; (ii) that the suicide/attempted suicide reported took place during the lockdown period, i.e., between 24 March to May 3; and (iii) the incident reported in the article had occurred in India. The criteria for excluding an article were: (i) the absences of unique identifiers and (ii) if the suicide/attempted suicide took place before or after the period of 24 March to 3 May. Two researchers then read through these relevant articles to identify duplicates (referring to the same individual who had died by suicide/attempted suicide) to arrive at a final number of unique individuals who had died by suicide or attempted suicide. An identical search strategy was used to find suicide and attempted suicide cases for the same dates (24 March to 3 May) in 2019.

Each article was then read by two researchers thoroughly to extract demographic data that included the name of the person, age, gender, occupation, marital status and education background. Other details were gathered on the location of the suicide or attempted suicide, the state or union territory where it occurred, the method, for example, if it was a death by hanging or the person consumed a toxic substance, as well as if it was a case of a suicide pact or homicide-suicide. A set of triggers/causes were identified from these news reports, categorised and then coded. To ensure uniform coding of triggers/causes across the data, two researchers coded them separately. Final ratings for any discordance was decided by the two researchers through a consensus meeting with the senior authors (LV and SP). The process of categorising and coding triggers/causes for both years was iterative and involved revisiting reports and reviewing statements from families and other official sources to capture the range of reasons that were attributed to the person’s suicide or attempted suicide. When coding the articles, cases were coded based on previously identified triggers/causes. Simultaneously, new and recurring triggers/causes were identified and listed. Any triggers/causes mentioned in an article quoted either from the suicide note, statement from family members, neighbour or colleagues of the person who died by suicide or attempted suicide or from statements made by investigating police officers were included as a trigger/cause. For each individual case, we coded and recorded as many triggers/causes reported in the article, because multiple triggers/causes were identified in many news reports.

Further, to distinguish between cases that were reported as a consequence of the COVID-19 crisis, the researchers also classified news reports in 2020 into three categories—‘COVID-19 related’ (if the reports mentioned fear of having COVID, fear of lockdown or were quarantined as a cause for suicide or attempted suicide), other COVID-19 issues were alcohol related (due to unavailability of alcohol during lockdown), police humiliation/brutality and social discrimination, ‘Non-COVID-19 related’ (for reports where this was not mentioned as a cause) and ‘Reasons yet to be ascertained’ for instances where no information was available, the cause was unclear, other triggers were not mentioned or if the case was still under investigation.

### Statistical analysis

All data are presented as categorical variables and summarized as frequency counts and percentages. Demographic variables and methods used for suicide were compared for suicide and attempts cases between the 2 years using Chi-squared (χ^2^) tests. Statistical significance for differences was set at p < 0.05. Analyses were performed using STATA software for Windows (version 14.2).

## Results

For the year 2020, the search identified 27,997 Google hits with 1095 suicide related articles for the relevant lockdown dates. Two researchers then read through these 1095 articles from 56 publications and removed 713 articles (referring to the same person who had died by suicide or attempted suicide) leaving a final total of 382 unique individual cases of which 326 were deaths by suicide, 43 attempted suicides and 13 undetermined cases. Cases were classified as undetermined if official sources were yet to ascertain whether the person died by suicide or it was a potential case of murder or accidental death.

**Fig. 1:**
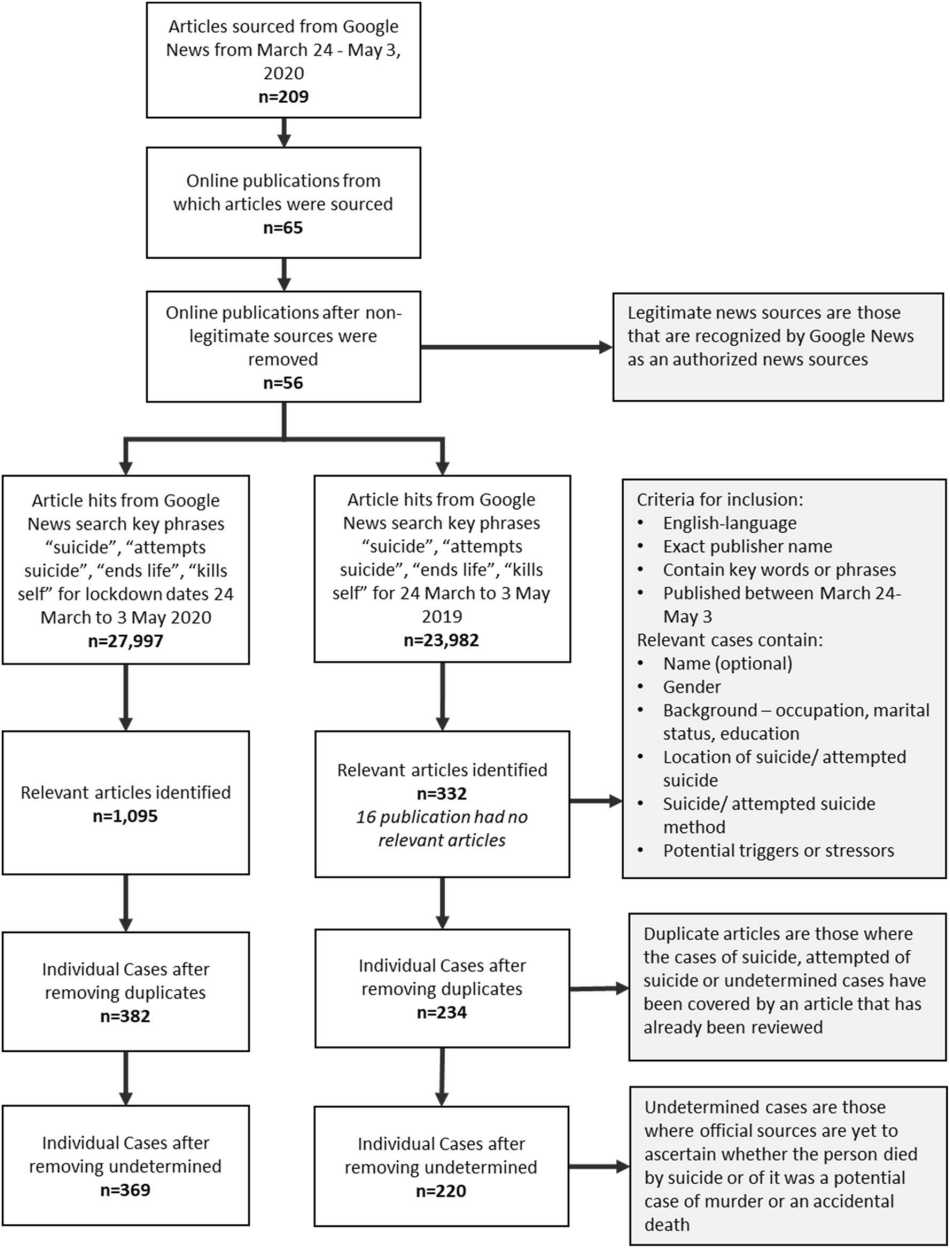
PRISMA chart

In 2019, of the 56 publications, there were no relevant articles in 16 publications. This search resulted in 332 relevant articles from 23,982 search results. After removing 98 articles with repeated stories about the same individual, we arrived at 234 unique cases, 196 were cases of death by suicide, 24 cases of attempted suicide and 14 undetermined cases of suicide (Fig. 1). Undetermined cases were not included in the analysis. The large difference in relevant articles between the 2 years (1095 in 2020 vs 332 in 2019) could be partially because a higher number of cases were frequently reported by multiple publications. Also, it should be noted that for the 16 publications for which no relevant articles were available in 2019, those publications generated 304/1095 articles in 2020, which is a significant number of articles.

Total number of suicides and attempted suicides reports in English language news increased by 67.7% from 220 such reports in 2019 to 369 reports during the lockdown in 2020.

We analysed the cases reported in news publications by year (comparing 2019 to 2020) and also compared COVID vs non-COVID cases during the lockdown.

### Year-wise comparison

There were significant demographic differences for suicide reports between the 2 years. Compared to 2019, suicides reported during lockdown were by older individuals, more likely to belong to age groups of 31–50 years vs < 30 years in 2019 (χ^2^ 8.2, p < 0.05), significantly more men’s suicides were reported during the lockdown (71.2% vs 58.7%, χ^2^ 8.4, p < 0.01), suicide cases were significantly more likely to be married (77.7% vs 49%, χ^2^ 28, p < 0.01) and suicides during lockdown were significantly more likely to be employed (82.9% vs 59.5%, χ^2^ 21.1, p < 0.01). (Table 1). Data on education was available only for 59 suicide and 7 attempts cases and hence not included in the analysis.

**Table 1:** Demographic characteristics of attempted and suicide cases in the year 2019 and 2020

Variables	Suicide		Attempts	
	2019 (n = 196)	2020 (n = 326)	2019 (n = 24)	2020 (n = 43)
Gender				
Male	115 (58.7)	232 (71.2)**	13 (54.2)	32 (74.4)
Age groups (years)	(n = 165)	(n = 298)	(n = 17)	(n = 32)
< 30	102 (61.8)*	146 (49)	9 (52.9)	10 (31.2)
31–50	38 (23)	104 (34.8)*	6 (35.2)	21 (65.6)
> 50	25 (15.1)	48 (16.1)	2 (11.8)	1 (3.1)
Marital status	(n = 147)	(n = 157)	(n = 12)	(n = 12)
Currently married	72 (49)	122 (77.7)**	6 (50)	10 (83.3)
Never married	69 (46.9)**	30 (19.1)	6 (50)	2 (16.7)
Divorced/separated/widowed	6 (4.1)	5 (3.1)	–	–
Occupation	(n = 121)	(n = 193)	(n = 14)	(n = 16)
Employed	72 (59.5)	160 (82.9)**	9 (64.3)	13 (81.3)
Unemployed	49 (40.5)	33 (17.2)	5 (35.7)	3 (18.7)

Values are presented as number of cases (%). Significant differences between both the years for suicide cases (gender: χ^2^ 8.4; age groups: χ^2^ 8.2; occupation status χ^2^ 21.1; marital status: χ^2^ 28.0)

*p < 0.05; **p < 0.01

There were significant differences between the 2 years for methods used for suicide and attempted suicides reports. During the lockdown in 2020, significantly more suicides were reported by hanging (64.4% vs 42%), fewer cases of poisoning (8.5% vs 21.5%) and fewer cases of jumping in front of a train (2% vs 9.4%) (χ^2^ 37.8; p < 0.05). Similarly, there were more attempted suicides using hanging (13.8% vs 0%), jumping from a height (22.2% vs 13%) and less by consumption of poison (22.2% vs 39.1%) and jumping in front of a train (0% vs 13%) however, the differences were not significant (Table 2).

**Table 2:** Methods used for attempts and suicide in the year 2019 and 2020

Methods	Suicide		Attempts	
	2019 (n = 181)	2020 (n = 295)	2019 (n = 23)	2020 (n = 36)
Consumption of poisonous substance	39 (21.5)	25 (8.5)	9 (39.1)	8 (22.2)
Drowning	10 (5.5)	11 (3.7)	1 (4.3)	4 (11.1)
Hanging	76 (42)	190 (64.4)*	–	5 (13.8)
Jumping in front of train	17 (9.4)	6 (2)	3 (13.0)	–
Jumping from height	20 (11)	30 (10.1)	3 (13.0)	8 (22.2)
Others (Self-immolation, self-infliction,)	19 (10.5)	33 (11.2)	7 (30.4)	11 (30.5)

Data not known for 46 suicide cases; 8 attempted cases. Values are presented as number of cases (%). Significant differences for method of suicides cases between 2019 and 2020 χ^2^ 37.8

*p < 0.05

There were significant regional differences in suicide reports between the 2 years. Bihar showed the highest increase (7 times) in news reports of suicide during the lockdown as compared to 2019.

Southern states namely Telangana, Tamil Nadu showed an increase in news reports of suicides between (2.6–50%) whereas in Kerala reports increased 4 times, Karnataka increased 2.5 times, while Andhra Pradesh and Puducherry showed reduced news reports of suicides.

In northern states namely Uttar Pradesh, Haryana, Chandigarh, Himachal Pradesh, Rajasthan, Jammu and Kashmir news reports of suicides increased by ~ 2–7 times whereas Punjab and Uttarakhand showed an increase between 26–50% and Delhi showed a decline in reported suicides.

Amongst other eastern and western states like Odisha and Maharashtra, news reports of suicides increased by 26–50% times whereas for central states like Jharkhand and Madhya Pradesh news reports increased from 2 to 7 times while they remained constant for Gujarat and Chhattisgarh between the 2 years.

Furthermore, we assessed presence and absence of triggers and compared between 2019 and 2020. With respect to the triggers as reported by online news media for suicides and attempted suicides, while there was no difference in reported history of mental illness between the 2 years (n = 15 in 2019 vs n = 18 in 2020) (p > 0.05), poor mental health was significantly more likely to be reported as a stressor/cause in 2020 in news articles for suicides (23.6% vs 15.3%, χ^2^ 5.2, p < 0.05) and attempted suicides (44.2% vs 4.2%, χ^2^ 11.8, p < 0.01). Poor physical health was also more likely to be reported in 2020 for suicides (14.1% vs 5.6%, χ^2^ 9.0, p < 0.01) and attempted suicides (14% vs 0%, χ^2^ 3.7, p = 0.05 marginally significant) while sexual harassment was less likely to be reported in 2020 (2.5% vs 7.7%, χ^2^ 7.8, p < 0.01).

### Relationship with COVID-19 illness

In the year 2020, news reports of 128 suicide cases and 29 attempted cases (Total = 157) mentioned fear of COVID, lockdown and/or the person was quarantined as a cause for suicide/attempted suicide.

These 157 cases were compared to 101 non-COVID suicides and attempted suicide cases where COVID was not associated clearly as a factor from the news reports. For the remaining cases (n = 111) the causes were unascertained and hence not included in the analysis.

COVID-19 cases of reported suicides and attempts were significantly more likely to be men (84.7% vs 60.4%, χ^2^ 19.4, p < 0.01), older (31–50 years 52.9% vs 25.8%, χ^2^ 17.3, p < 0.01) and employed (91.5% vs 64.3%, χ^2^ 17.1, p < 0.01) (Table 3), while there were no difference between the two groups on methods used for suicide/attempted suicide. Higher number of COVID cases were reported to have poor mental health (40.1% vs 20.8%, χ^2^ 10.4, p < 0.01) and poor physical health (24.8% vs 7.9%, χ^2^ 11.8, p < 0.01).

**Table 3:** Comparison of COVID and non-COVID cases year 2020

	COVID (n = 157)	non-COVID (n = 101)	Chi-squared test significance
Gender			
Male	133 (84.7)*	61 (60.4)	19.4; p < 0.05
Age groups (years)	(n = 138)	(n = 89)	
< 30	45 (32.6)	51 (57.3)	
31–50	73 (52.9)*	23 (25.8)	17.3; p < 0.05
> 50	20 (14.5)	15 (16.8)	
Marital status	(n = 54)	(n = 67)	
Currently married	41 (75.9)	51 (76.1)	1.0; p > 0.05 NS
Never married	10 (18.5)	15 (22.4)	
Divorced/separated/widowed	3 (5.6)	1	
Occupation	(n = 94)	(n = 56)	
Employed	86 (91.5)*	36 (64.3)	17.1; p < 0.05
Unemployed	8 (8.5)	20 (35.7)	

Values are presented as number of cases (%)

A significant difference was also observed between COVID and non-COVID cases for alcohol withdrawal-related suicides and attempted suicides (24.2% vs 0%, χ^2^ 28.6, p < 0.01).

## Discussion

The current study is based on online news media reports of suicide and attempted suicide between 24 March to 3 May 2020 compared to the suicides reported in same news publications for the same dates in 2019 in India. There were 369 news reports of suicides and attempted suicides in 2020 as compared to 220 in 2019, a 67.7% increase in reported suicides and attempted suicides during the lockdown. This increase may reflect an increased awareness in Indian media and journalists about the impact of major psychosocial disruptions like a lockdown on suicides and attempted suicides or it may reflect more sensational reporting by Indian media during the pandemic crisis. Alternatively, there exists the possibility that these increased reports reflect a true increase in suicides and attempted suicides in the community. However, in the absence of any official data on suicides and attempted suicides, we are unable to ascertain if this is the case.

There is a significant change in the demographic factors of reported suicidal behaviour between 2019 and 2020. During the COVID lockdown, there were significantly more reports of suicides and attempted suicides by older employed men. It is difficult to attribute these demographic changes in news reports merely to changes in journalists’ reporting behaviour as nothing has happened recently to significantly change journalists reporting styles across the country. On the other hand, if these news reports are representative of suicides and attempted suicides in the community, it is a worrying sign as it could indicate the impact of economic crisis due to COVID lockdown and possible job losses. The Centre for Monitoring Indian Economy estimated that 27 million young people between the ages of 20–30 years lost their jobs in April 2020 during the lockdown [11]. It is also estimated that nearly 100 million Indian jobs are at risk in the coming months [12]. While modelling studies have predicted significant increases in suicides and attempted suicides in other countries [13, 14] in the coming years as unemployment increases, our data raises the possibility that the profile of suicides and attempted suicides may be changing in India during the COVID pandemic. This is also reflected in our data on ‘COVID related’ (fear of catching COVID, fear of lockdown or in quarantine for suspected or actual COVID diagnosis) suicides and attempted suicides where news reports show a preponderance of employed men aged 30–40 years.

Recent research on Google Trends reveals that search volumes for financial and work-related issues have increased [15]. The economic consequences of the pandemic are likely to lead to an increase in suicides as shown during past economic crises [16].

There were significant differences in the reported suicide methods in the 2 years. During the lockdown, more suicides are reported by hanging and there were fewer cases of poisoning and jumping in front of a train.

A systematic review of self-poisoning with pesticides estimated that there were ~ 110,000 pesticide suicides each year (13.7% of global suicide) worldwide [17]. Ingestion of pesticide is a common and lethal means of suicide in India. During the lockdown, all shops were closed, and majority of farming activities were suspended, thus reducing access to pesticides which may be reflected in the lower number of suicides by poisoning during this period. Similarly barring a few trains transporting goods, all train services were stopped. This may have reflected in the significant reduction of suicides by jumping in front of a train.

A consistent finding from past studies from India is that southern states have a high suicide rate and the northern states have significantly lower than national rates [7, 18]. The current study shows that news reports reflect this trend, with higher suicides reports from southern states. However, it is interesting that news reports of suicides increased in 2020 in states which traditionally have low suicide rates such as Bihar, Uttar Pradesh, Rajasthan, Haryana, and Chandigarh. News reports of suicide from Bihar [19] which had one of the lowest suicide rates in the country at 1.2/100, 000 in 2018, has shown an almost 7 times increase in news reports of suicide and attempted suicide in 2020 compared to 2019. These states are economically less developed with inadequate health infrastructure which may render people in these states more vulnerable to suicides in the COVID pandemic. Long term studies are needed to determine whether these traditionally low suicide states are likely to witness an increase in their suicide rates.

Significant number of news reports of suicides and attempted suicides in 2020 had poor mental health and poor physical health. This may reflect an increasing awareness amongst journalists about COVID-19’s impact on mental and physical health and the role of mental and physical health issues in suicidal behaviour. Alternatively, it raises the possibility that those who are both physically and psychologically vulnerable are at a higher risk of suicide. The finding of significantly increased reports of poor mental health and poor physical health, in news reports may reflect the health effects of the lockdown on those with no previous mental or physical health conditions. Access to health services for conditions other than COVID were significantly negatively impacted in India [20] during the lockdown and this may have contributed to poor physical health and mental health.

The reduction in news reports of sexual harassment as a cause of suicide and attempted suicides compared to last year may reflect that almost the entire Indian population was housebound during the lockdown with little possibility of any social interaction. However, we are surprised with the lack of reports of domestic violence as predisposing to suicides and attempted suicides in both years. This may either reflect the general under-reporting of gender-based violence in news or may be a reflection that most cases of reported suicides are by men and hence gender-based violence was not a factor in reporting.

An unusual feature of news reports of suicides during the lockdown are 39 alcohol-related suicides and 7 attempted suicides as compared to no such suicide/attempted suicide cases in 2019. 34 of the 39 suicides were reported in the first two weeks of the lockdown (26 March 2020 to 5 April 2020), 4 cases in the subsequent 2 weeks (6 April 2020 to 19 April 2020) and the last such case was reported on 25 April 2020. As part of the lockdown imposed by the Government on 24 March 2020, all shops including those selling alcohol were shut down, resulting in people going into unplanned alcohol withdrawal in the two weeks after the lockdown was imposed [21]. As time progressed, there was recovery from alcohol withdrawal and these suicides gradually reduced to zero for the rest of the lockdown period. Traditionally reduced alcohol availability and consumption reduces the suicide and attempted suicides. However, these alcohol withdrawal suicides during lockdown may be due to abrupt closure of all bars and restriction on the sale of alcohol, pushing people into unplanned alcohol withdrawal and restricted access to health services.

There are many limitations to our study. First, we only searched for suicide and attempted suicide reports in English language news publications and therefore may have missed reports appearing exclusively in Indian language publications (India officially recognizes 22 languages with many hundreds of publications in Indian languages and not all are available online). Second, India has nearly 300–500 suicides each day and only a small fraction (2–3%) of these get reported in news publications. Hence an increase in news reports of suicides or attempted suicides may not directly correlate with an actual increase in suicides and attempted suicides but may only be due to increased reporting. It is entirely possible that journalists and news publication editors have become more sensitive to mental health and suicide-related issues during COVID lockdown and this is reflected in more reports being published. Finally, news reports are not systematic psychological autopsy reports and the information quality in these news reports are likely to be variable and not standardized.

The strength of the study is that we reviewed all the major English newspapers with maximum readership covering the entire country. Two researchers independently reviewed and triaged the data to minimize bias. Despite the limitations, we believe our findings highlight important points which may provide guidance to policymakers in India for suicide prevention in the coming months. There is a need for collecting and maintaining statistics on suicides and attempted suicides nationally as well as regionally and the current system of data collection by the NCRB needs significant improvement. Such data also needs to be made available to researchers to study trends and help policymakers make better decisions for suicide prevention.

## Conclusion

These findings raise the possibility that the pandemic may have increased the risk of suicide in employed middle-aged men even during the early phase, and a likelihood of further increases during the economic downturn which is expected to follow. However, community data on suicides and attempted suicides is needed to confirm this possibility. It is therefore imperative that the Government of India examines the NCRB data on suicides during this period and also release this data to researchers for further examination. If our findings are confirmed by the community data collected by NCRB, governments in India (both federal and state governments) need to prioritise addressing the economic fall-out of COVID pandemic to also address suicide prevention. The increase in alcohol-related suicides reports points to the need for a national alcohol policy to reduce access to and availability of alcohol as suggested by the World Health Organization as an important universal suicide prevention strategy. Vulnerable groups like migrant workers, elderly and youth need to be assessed for suicide risk and provided with adequate psychosocial support. The COVID pandemic also provides us with an opportunity for cross-sectoral collaboration for suicide prevention rather than restricting suicide prevention to the health sector.

## Data Availability

The datasets will be made available to appropriate academic parties on request from the principal investigator in accordance with the data sharing policies of the institute.

## Abbreviations

NCRB: National Crimes Record Bureau
USA: United States of America
COVID-19: Coronavirus disease
